# Local and global modes of drug action in biochemical networks

**DOI:** 10.1186/1472-6769-9-4

**Published:** 2009-04-07

**Authors:** Jean-Marc Schwartz, Jose C Nacher

**Affiliations:** 1 Faculty of Life Sciences, University of Manchester, Manchester M13 9PT, UK; 2 Department of Complex Systems, Future University-Hakodate, Hokkaido 041-8655, Japan

## Abstract

**Background:**

It is becoming increasingly accepted that a shift is needed from the traditional target-based approach of drug development towards an integrated perspective of drug action in biochemical systems. To make this change possible, the interaction networks connecting drug targets to all components of biological systems must be identified and characterized.

**Results:**

We here present an integrative analysis of the interactions between drugs and metabolism by introducing the concept of metabolic drug scope. The metabolic drug scope represents the full set of metabolic compounds and reactions that are potentially affected by a drug. We constructed and analyzed the scopes of all US approved drugs having metabolic targets. Our analysis shows that the distribution of metabolic drug scopes is highly uneven, and that drugs can be classified into several categories based on their scopes. Some of them have small scopes corresponding to localized action, while others have large scopes corresponding to potential large-scale systemic action. These groups are well conserved throughout different topologies of the underlying metabolic network. They can furthermore be associated to specific drug therapeutic properties.

**Conclusion:**

These findings demonstrate the relevance of metabolic drug scopes to the characterization of drug-metabolism interactions and to understanding the mechanisms of drug action in a system-wide context.

## Background

There is a growing perception that the traditional approach for drug development has not been as effective as could be expected [1]. Although investments by pharmaceutical companies have been growing continuously in the last decades, the number of newly approved drugs has not followed the same trend [2,3]. Currently the main trend in drug design and development follows the target-based approach. The drug target is generally a single gene or gene product which has been clearly identified as having an action on the disease. Disease treatment seeks to modulate this action without affecting other processes in the organism. However, many diseases are multifactorial, and the current approach fails to take such systemic aspects into account [4-6]. The complex interactions between numerous molecular processes and pathways involved in diseases imply that any treatment targeting a local element will create secondary effects on the entire system. The lack of an integrative investigation of drug action can lead to either positive or negative systemic effects being missed. In the first case, the treatment only provides limited and localized cure for the disease while failing to address large-scale causes. In the second case, new drugs are developed with harmful side effects that are only revealed at a late stage of clinical studies. This problem of drug attrition is a major concern for the pharmaceutical industry and an important source of financial costs. Additional problems can be caused by side-effects resulting from combinations of several drugs, arising from unexpected interactions which were not revealed by local analysis.

To increase the productivity of drug development and the efficiency of new drugs, there is thus a strong need to move beyond the investigation of drug action from the point of view of direct targets only, and to take into account the full network of interactions connecting the targets with the rest of the system. Drug development needs to be approached not only in terms of a molecular biology process, but also as a systems biology process [7-10]. This awareness has recently led to the completion of major studies revealing the large-scale interactions between drugs, drug targets and diseases from a network-based perspective [11-15]. However, these approaches did not unveil yet how specific drugs interact in biochemical systems, and new methods are needed to gain an integrated perspective of the mechanisms of drug action in complex systems.

To this aim, we here introduce a new view of drug-metabolism interactions based on the concept of *metabolic drug scope*. The concept of a *scope *was previously developed in metabolic networks [16]. It uses an expansion process based on the principle that, for any reaction to take place, all necessary substrates must be present and the products of these reactions may in turn be used by other reactions. Starting from a set of seed compounds, reactions whose substrates are available in the seed set are iteratively added, resulting in the generation of a series of expanding networks. When no further reaction can be added, the final network obtained is defined as the *scope *of the seeds. The scope represents the set of all compounds that can in principle be synthesized from the seeds. The properties of scopes in metabolic networks have been described in great detail [17-19]. This concept was already proven valuable in revealing features of the evolution of metabolic systems, leading notably to demonstrating how the shift from anoxic to oxic environments transformed the architecture of metabolic networks and how the availability of oxygen allowed an increase in biological complexity [20]. Recently, it was shown that features of scopes could also be linked to chemical structures and biological functions of the seeds [21].

We here extend the scope paradigm to drug-metabolism interactions. Many drugs target genes or gene products that are involved in metabolic functions. When a metabolic reaction is targeted by a drug, the concentrations of its substrates and products are affected as a result of drug action. Other reactions which use the products of the initial reactions are in turn affected by these perturbations. By iterative expansion, the entire *scope *of the initial substrates and products can in principle be affected by the drug. Basing on this principle, we thus introduce the concept of a *metabolic drug scope *defined by the following: the scope of a drug is the scope resulting from the expansion of a set of seed compounds containing the substrates and products of all metabolic reactions targeted by that drug.

The metabolic drug scope essentially represents the largest network of possible action of a drug in a metabolic system. In the following sections, we show how metabolic drug scopes reveal new features of the systemic modes of drug action through a detailed analysis of the scopes of 276 human approved drugs from the DrugBank database [22] having metabolic targets. As scopes depend on the topology of the metabolic network used in the expansion process, we analyzed the action of each drug in four different systems: a human vs. a reference metabolic network consisting of the union of all organisms, and for each of those a reversible vs. an irreversible topology. Further details on the construction of metabolic drug scopes are provided in the Methods section. Our results show that the distribution of scopes is highly uneven and that they can be classified into different categories. Some drugs have small scopes associated to localized action, while others have large scopes associated to potential widespread systemic action. These classes are relatively well conserved throughout the four systems. A network of metabolic drug scopes, where scopes are connected when their Jaccard distance is smaller than a given threshold, confirms these characteristics, and a k-core decomposition algorithm reveals several highly connected central components as well as peripheral subgraphs. We subsequently analyzed the correlations between metabolic drug scopes and therapeutic properties of drugs, and show that therapies are not distributed uniformly but can often associated to specific groups of scopes, correlated to similar k-cores. Metabolic drug scopes therefore offer a new avenue to analyze drug-metabolism interactions and may provide valuable assistance to the drug development and assessment process.

## Results

### Metabolic drug scopes are highly uneven

**Figure 1:**
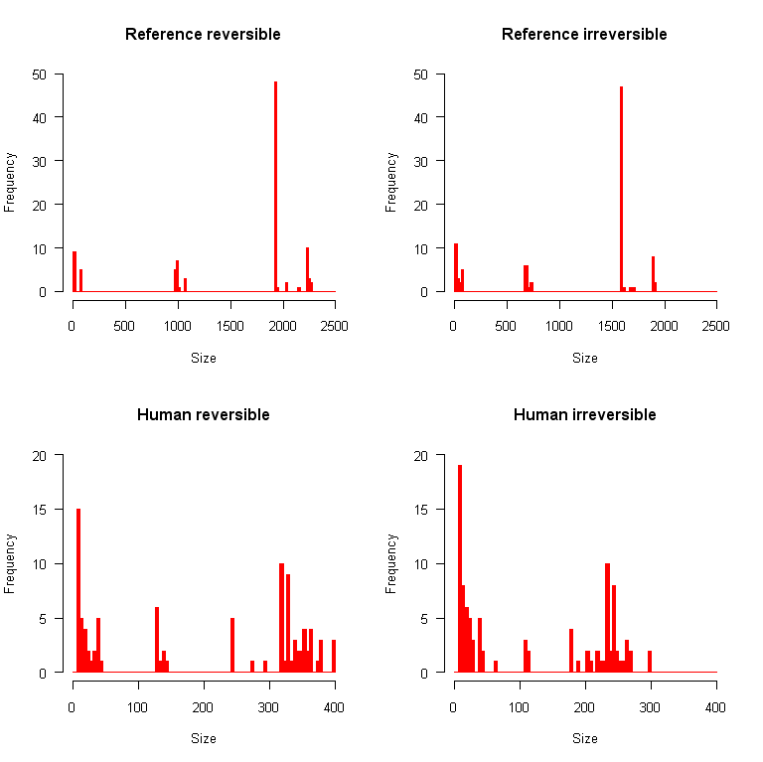
**Distribution of scope sizes**. Multiple identical scopes are counted only once. The width of intervals is 20 for reference networks (top), 5 for human networks (bottom).

The first step in characterizing the properties of metabolic drug scopes consisted in analyzing the distribution of their sizes. In many cases multiple drugs have the same targets and these drugs automatically have the same scope. To avoid identical scopes to be counted several times, we beforehand selected a subset of scopes where only one representative of each group of identical scopes was kept, resulting in a group of 97 scopes which are all different. The distributions of sizes of these scopes are shown in Figure 1 for each of the four metabolic networks.

Histograms reveal a highly heterogeneous distribution. Scope sizes are not distributed evenly, nor do they show any kind of regular distribution law. On the contrary, sizes are often concentrated around particular values: for example, in the reference reversible network there are 48 scopes of size comprised between 1920 and 1940. The presence of such peaks can be attributed to particular metabolites. It is known that the inclusion of ubiquitous metabolites has a strong influence on the size of scopes [23]. The most frequent of them is ATP, which together with water and oxygen generate a scope of 1929 compounds. The previously mentioned peak is thus clearly attributable to the presence of ATP. Nevertheless, it is remarkable that scopes do not exhibit larger variations around peaks. This observation suggests that metabolic drug scopes can be separated into distinct and relatively homogeneous groups, where internal variations are small.

We analyzed the distribution of scope sizes with regard to the total number of targets of each drug, including non-metabolic targets, in the four metabolic networks (Additional File 1). The distribution shows that there is no significant correlation between these two variables (correlation coefficients between 0.15 and 0.19). This observation confirms that scopes are more dependent on the presence of particular metabolites than on the number of targets.

**Figure 2:**
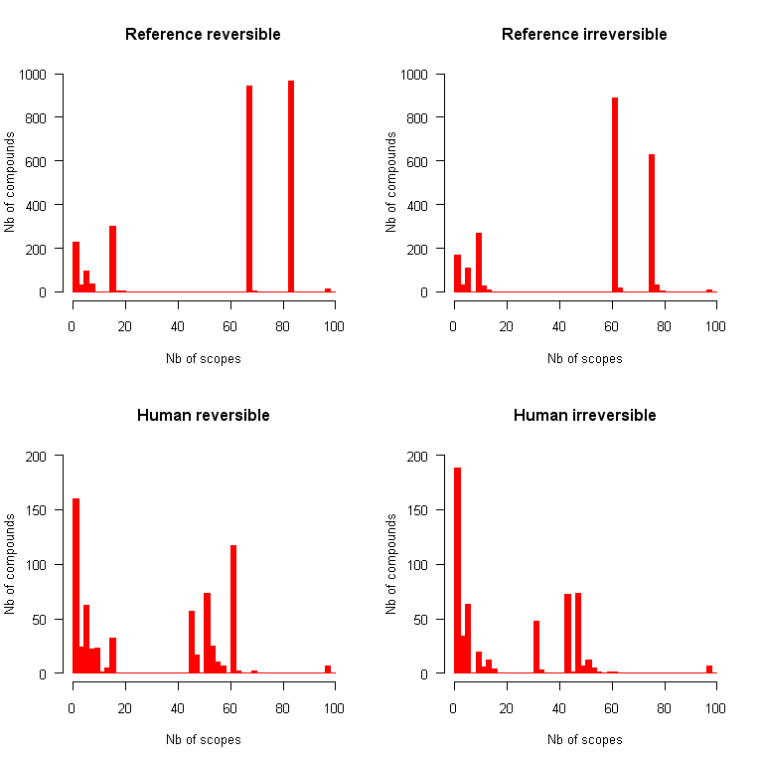
**Frequencies of occurrence of compounds in scopes**. Multiple identical scopes are counted only once. The width of intervals is 2.

**Figure 3:**
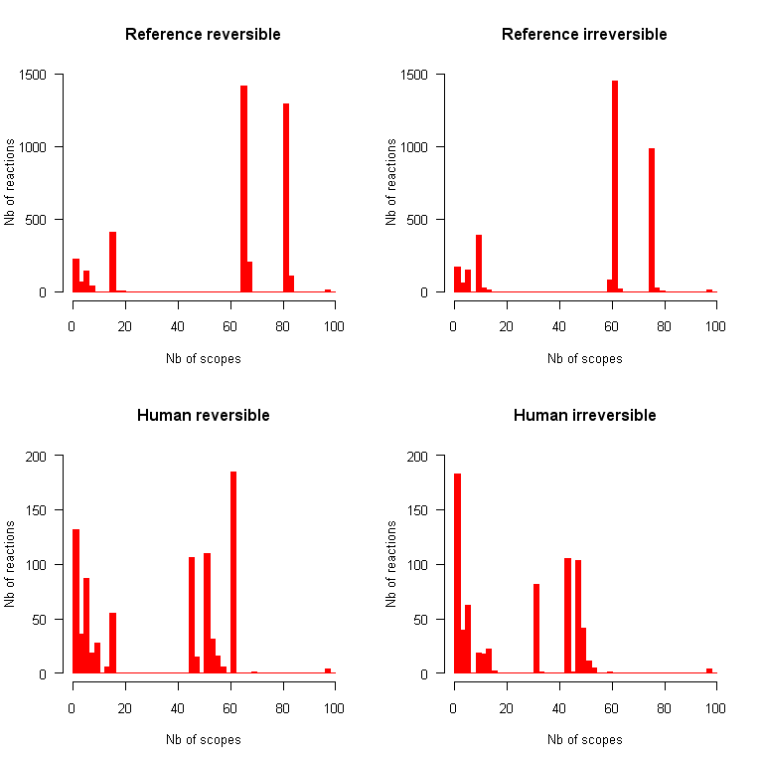
**Frequencies of occurrence of reactions in scopes**. Multiple identical scopes are counted only once. The width of intervals is 2.

The frequencies of occurrence of chemical compounds and reactions in metabolic drug scopes are plotted in Figures 2 and 3 respectively. These distributions are very irregular and do not follow any classical law either. Peaks can be observed at some values, separated by intervals of zero frequency. Such distributions can be explained by the fact that scopes tend to grow through the incorporation of groups of metabolites, ubiquitous metabolites bringing large numbers of other metabolites with them. The sharp peaks observed at some high values, particularly for the reference network, are a further indication that groups of very similar scopes exist. Each of these peaks corresponds to a group of scopes containing many of the same compounds and reactions. It was thus desirable to use clustering methods to reveal such groups of drugs.

### Scopes reveal four main categories of systemic drug action

**Figure 4:**
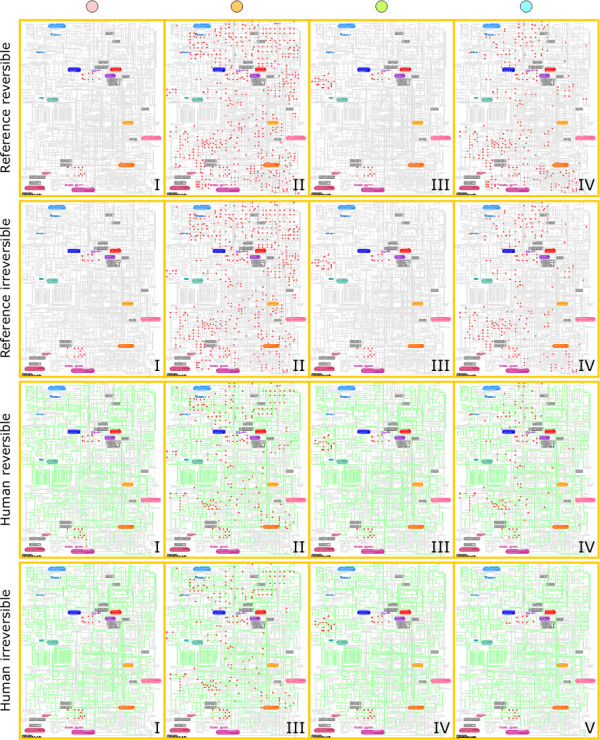
**Consensus scopes of drug groups plotted over a Kegg Atlas genome scale metabolic map for the reference reversible metabolic network**. Chemical compounds belonging to the scope appear as red dots.

We clustered metabolic drug scopes using the Jaccard distance as a measure of dissimilarity between scopes (see Methods). In the reference reversible network, four clusters of metabolic drug scopes were obtained, whose main properties are listed in Table 1. In order to highlight the main features and differences between these clusters, we constructed the *consensus scope *of each cluster. The consensus scope is the set of compounds present in at least 50% of all scopes of a cluster (see Methods). Consensus scopes of the four clusters are plotted over a genome scale metabolic map in Figure 4.

**Table 1:** Clusters of metabolic drug scopes in the reference reversible network.

Cluster Id	Number of drugs in cluster	Average scope size	Significant compounds in consensus scope
I	37	15	-
II	163	1963	ATP, NAD+, Pyruvate, Galactose
III	35	69	Arachidonate
IV	41	995	Pyruvate, Galactose

Clear differences can be observed between these four drug clusters. Cluster I is composed of drugs whose scope is very small. These scopes only slightly differ from the minimal scope induced by water and oxygen alone. When only water and oxygen are used as seeds and cofactors are allowed, a minimal scope of 12 compounds is obtained. The average size of scopes in cluster I is 15, and the size of their consensus scope is 12. These scopes thus mainly consist of the minimal scope with the addition of a handful of other compounds. These additional compounds are isolated and poorly connected to the rest of the metabolic network, as they do not further increase the size of the scope. Drugs belonging to cluster I are therefore only able to affect a limited and specific part the metabolic network. Their action is not allowed to propagate beyond the intended target and should be mainly local.

Cluster III contains the same minimal component induced by water and oxygen, but additionally contains a larger component centered on arachidonic acid metabolism. This component, which can be seen at the left-hand side of the genome scale metabolic map (Figure 4), is absent from the three other clusters. Interactions of certain drugs with arachidonate metabolism have long been known and this pathway is particularly important in anti-inflammatory treatments [24,25].

Clusters II and IV on the contrary contain large scopes spreading through many parts of the metabolic network. Drugs belonging to these clusters can therefore in principle affect a wide area of metabolism and potentially have widespread systemic effects. Such dramatic increase in the size of scopes can be achieved by the inclusion of particular metabolites. For example, Raymond and Segrè [20] showed that the availability of oxygen leads to a major expansion of the size and complexity of metabolic systems. Although oxygen and water where assumed to be always available in our analysis, similar increases can be achieved by the inclusion of other metabolites. The most important of them is ATP, which explains the distinction between cluster II and IV: ATP is present in all scopes of cluster II but absent from the scopes of cluster IV. It is worth noticing that ATP does not have the largest scope of all metabolites though: this position is held by adenosine 5'-phosphosulfate [23], which is only present in a small number of scopes of cluster II (16 out of 163). The size of a compound's scope alone is thus not a determining factor for its recurrent occurrence in metabolic drug scopes. Pyruvate plays a major role too in separating different clusters, as it is present in clusters II and IV, but absent from clusters I and III.

### Drug categories are conserved in different networks

The scope construction process fundamentally depends on the topology of the underlying metabolic network. We therefore conducted the same analysis in four different networks: a reference network consisting of the union of all organisms vs. a human metabolic network, and in each of those a reversible vs. an irreversible topology (see Methods). Clustering of metabolic drug scopes in these other networks resulted in more clusters being found than in the reference reversible network (Tables 2, 3, 4). However, most of these new clusters are very small, containing only in a handful of drugs. They would fall into one of the four main clusters if the cutoff were increased. The detailed composition of clusters is provided for the four networks in Additional File 2.

**Table 2:** Clusters of metabolic drug scopes in the reference irreversible network.

Cluster Id	Number of drugs in cluster	Average scope size	Significant compounds in consensus scope
I	40	14	-
II	154	1606	ATP, NAD+, Pyruvate, Galactose
III	35	68	Arachidonate
IV	39	687	Pyruvate, Galactose
V	1	27	CoA
VI	3	47	-
VII	3	35	-
VIII	1	38	-

**Table 3:** Clusters of metabolic drug scopes in the human reversible network.

Cluster Id	Number of drugs in cluster	Average scope size	Significant compounds in consensus scope
I	77	9	-
II	94	339	ATP, Pyruvate, Tyrosine, Galactose
III	35	40	Arachidonate
IV	30	130	Pyruvate
V	12	244	Pyruvate, Tyrosine, Galactose
VI	8	21	-
VII	1	44	Tryptophan, Tyrosine
VIII	4	11	-
IX	5	21	-
X	3	22	-
XI	2	14	-
XII	2	11	-
XIII	1	30	-
XIV	1	33	Tyrosine
XV	1	16	-

**Table 4:** Clusters of metabolic drug scopes in the human irreversible network.

Cluster Id	Number of drugs in cluster	Average scope size	Significant compounds in consensus scope
I	62	8	-
II	24	10	-
III	85	230	ATP, Pyruvate, Galactose
IV	35	40	Arachidonate
V	14	11	-
VI	15	21	-
VII	13	27	-
VIII	5	110	Pyruvate
IX	8	21	-
X	1	12	CoA
XI	1	25	CoA
XII	2	53	Tryptophan
XIII	3	21	-
XIV	2	14	-
XV	1	16	-
XVI	2	11	-
XVII	1	24	-
XVIII	1	30	Tyrosine
XIX	1	16	-

**Figure 5:**
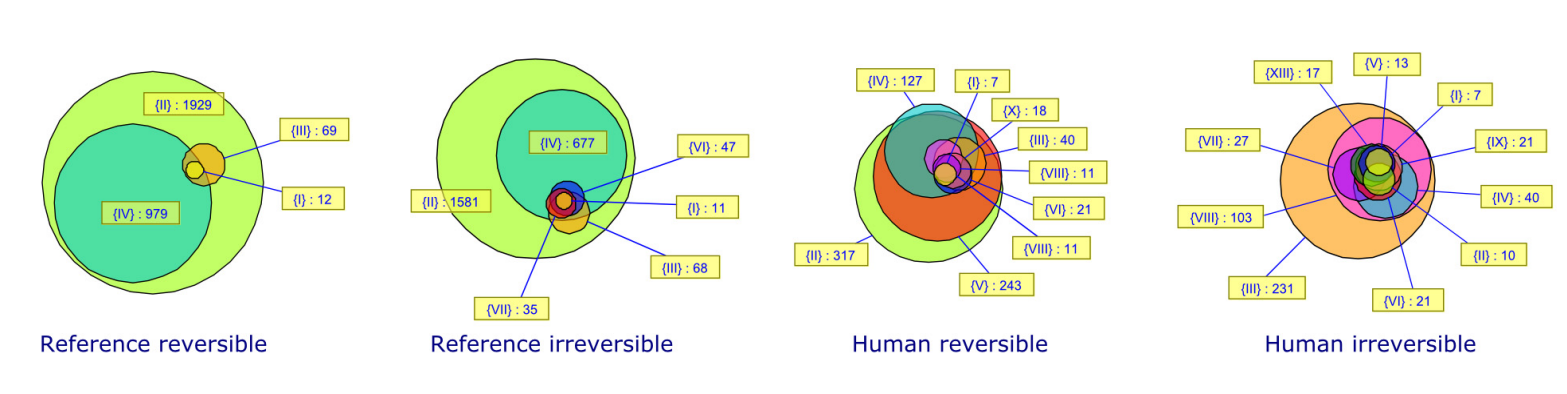
**Venn diagrams showing overlapping between consensus scopes of clusters in each network**.

The four main clusters are still present and exhibit similar characteristics as in the reference reversible network. Although the average size of scopes is smaller in irreversible and human networks than in the reference reversible case (which is natural because scopes in irreversible and human networks are necessarily subsets of scopes in the reference reversible network), it is worth noticing that the consensus scopes of the four main clusters remain very similar to their reference reversible counterparts (Figures 2, 3, 4). Overlaps between consensus scopes from different clusters were made apparent by Venn diagrams (Figure 5). Groups consisting of large (II and IV) and small scopes (I and III) are clearly distinguishable in the reference reversible network. In the reference irreversible network, this pattern is well conserved despite additional small clusters appearing. In human networks, more complex overlapping patterns emerge but two groups of large scopes are still present.

**Figure 6:**
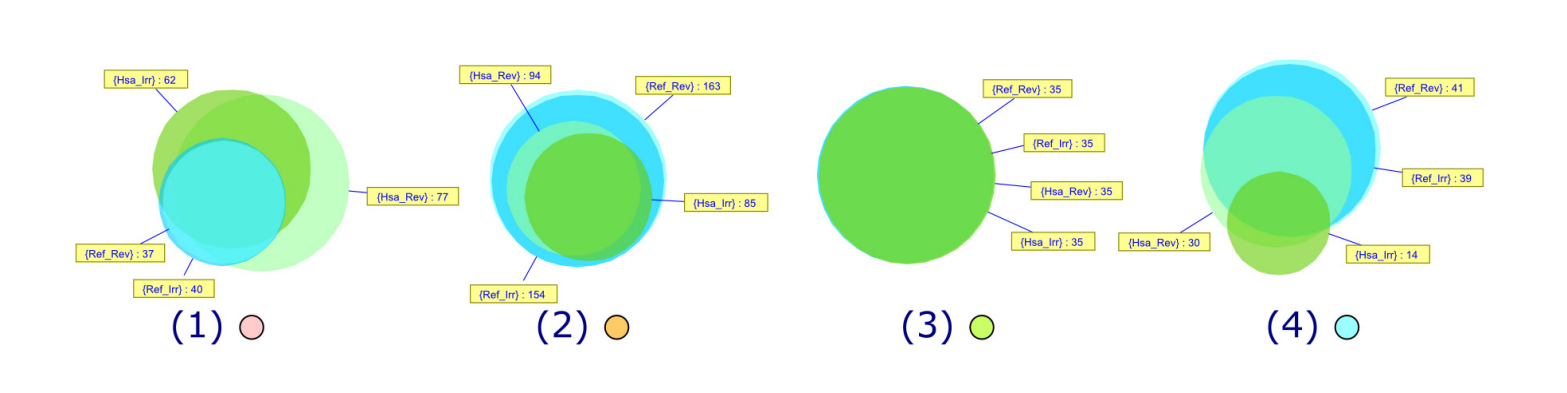
**Venn diagrams showing overlapping between drugs belonging to the four main clusters in each network**. (1) represents cluster I for all four networks. (2) represents cluster II for the reference reversible/irreversible and the human reversible networks, cluster III for the human irreversible network. (3) represents cluster III for the reference reversible/irreversible and the human reversible networks, cluster IV for the human irreversible network. (4) represents cluster IV for the reference reversible/irreversible and the human reversible networks, cluster V for the human irreversible network.

More significantly, the drug composition of the four main clusters is well conserved throughout the different networks (Figure 6). Cluster III is even 100% conserved in all four networks. The only significant difference appears with cluster IV, which is less conserved in the human irreversible network. The latter network is the less densely connected of all four, therefore emphasizing the dependence of metabolic drug scopes on local targets and leading to a higher scattering of scopes. Table 4 shows that this network has a large number of very small clusters, making it more difficult to identify large groups of drugs with common properties as a result. On the other side, this network is the most appropriate when it comes to identifying highly specific drug-metabolism interactions, as scopes tend to be more restricted to the immediate neighborhood of drug targets.

### Metabolic drug scopes are correlated to therapeutic properties

**Figure 7:**
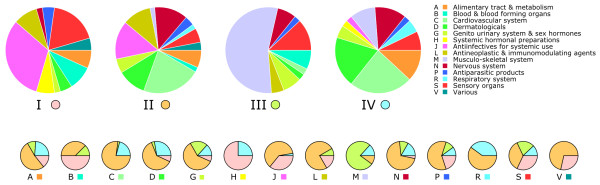
**Top: Pie charts showing the distribution of therapeutic classes in the four main groups of drugs**. Bottom: Repartition of each therapeutic class through the four groups. Colors correspond to identifiers I to IV as indicated below the large pie charts.

As metabolic drug scopes are related to features of the systemic modes of drug action, a fundamental question was to determine whether the properties of metabolic drug scopes could be linked to therapeutic properties of the drugs themselves. We consequently analyzed the distribution of therapeutic properties, defined by level 1 tags of the ATC classification (see Methods), in the four main groups of drugs identified by clustering of their scopes. As shown in Figure 7, the repartition of therapies is not uniform in the four groups. While clusters II and IV show relatively similar compositions in therapies, they together strongly differ from clusters I and III. 55% of drugs in cluster III belong to the musculo-skeletal system (M) category, and 77% of all class M drugs belong to cluster III. The enrichment of class M in cluster III is statistically highly significant (*p*-value = 10^-21^). Cluster I is dominated by antiinfectives (J; *p *= 3·10^-4^) and sensory organs drugs (S; *p *= 0.021); it contains half of all blood related drugs (B; *p *= 0.009) and three quarters of hormonal preparations (H; *p *= 0.002). Clusters II on the contrary is dominated by cardiovascular drugs (C; *p *= 0.039), this class is almost absent from cluster I and III.

The fact that clusters II and IV show more similarities than the other clusters must be put into relation with the fact that these two clusters correspond to large scopes, while clusters I and III correspond to small specific scopes. Therefore, there appears to be a relationship between the composition and extent of a metabolic drug scope and its therapeutic properties. This observation reinforces the potential role of scopes as an indicator of drug action and effects in a systems-wide context.

**Figure 8:**
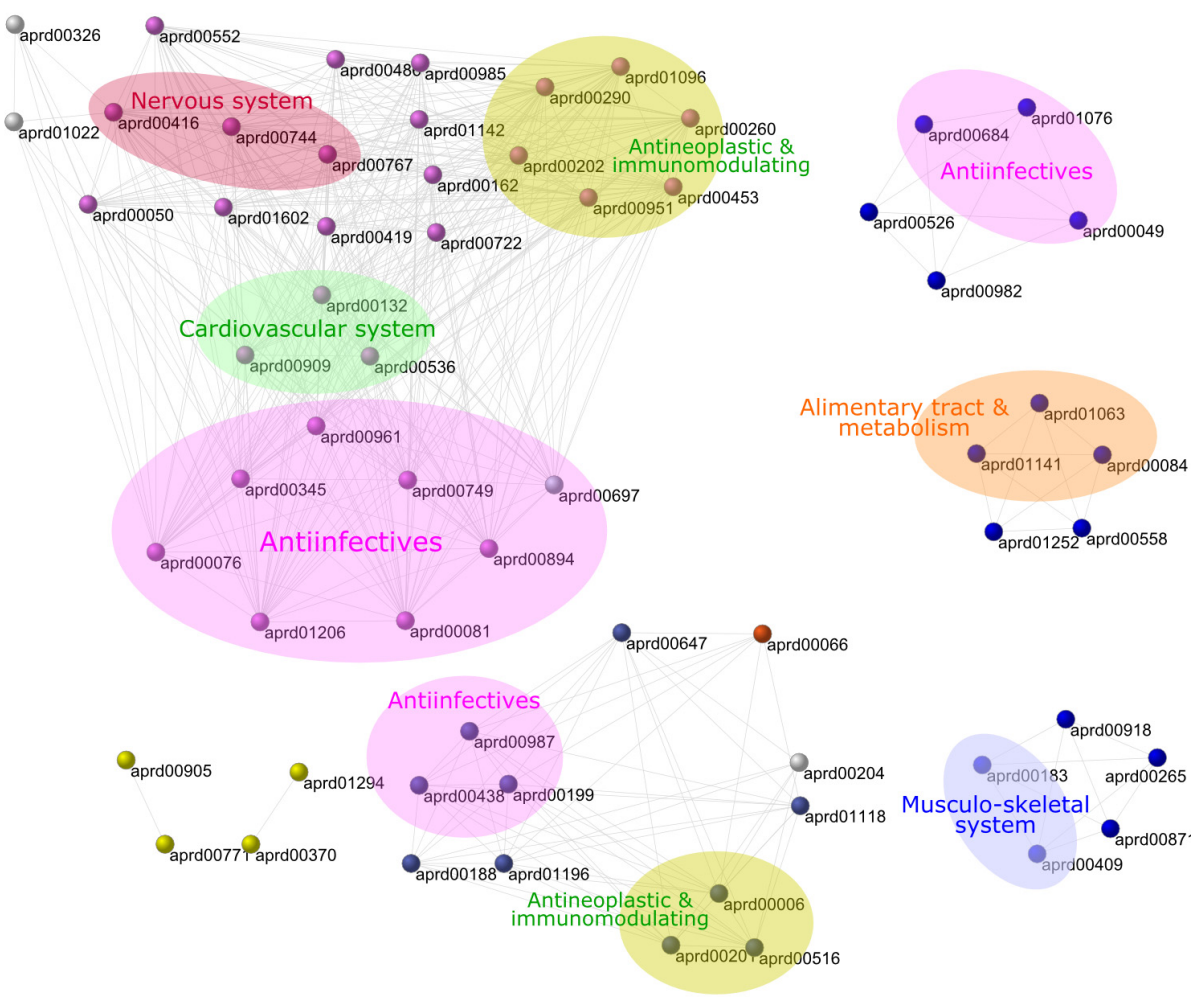
**Network of metabolic drug scopes in the human irreversible case**. Nodes of same color belong to the same k-core, isolated nodes are not shown. Groups of drugs belonging to the same therapy class are highlighted. Similar patterns were observed in the human reversible case.

A complementary analysis of the relationships between metabolic drug scopes and therapies was realized by constructing a network of scopes (see Methods). Complex interwoven interactions can be analyzed in networks by identifying the cohesive building blocks of the system. Cohesive subgraphs are sets of nodes with high density of edges in their network neighborhood. We applied an iterative decomposition method to uncover k-core subnetworks, defined as the largest subgraphs where every node has at least degree k. Figure 8 shows the decomposition of a network of metabolic drug scopes, for a threshold of 0.2 in the Jaccard distance between scopes and in the human irreversible case. Each colored node represents a different core partition. Highly connected drugs are located in innermost cores where overlapping with complete therapy graphs is more evident. The overlapping of therapy cliques with k-core subgraphs confirms links between the composition of scopes and the effects of drugs.

## Discussion

It is a widely accepted fact that new approaches are needed to facilitate and increase the quality of the drug target identification and drug development processes. High levels of investment by pharmaceutical companies in recent years have not been followed by a corresponding increase in discovery and commercialization of new drugs, and the traditional reductionist approach of drug development is seen as a possible cause for this lack of success. Many diseases are multifactorial and can not be handled by targeting only isolated molecules, but systemic aspects need to be taken into account. The toxicity and harmful side-effects of some drugs can also be accounted to by a failure of considering drug action in an integrated and systemic way. When a molecular target is perturbed by a drug, effects are not limited to that target but potentially extent to the whole interaction network connected to it. It therefore becomes more and more necessary to study drug action with a systems biology perspective, and important efforts are needed to construct and characterize the interaction networks connecting drug targets to all components of a biological system.

The metabolic network is one of these interaction networks, and metabolic drug scopes provide a straightforward and efficient tool to construct them and to analyze their properties. We have shown that metabolic drug scopes can vary greatly in size and distribution. When a drug has a small scope, its action is necessarily restricted to a small set of reactions and compounds. Whether this feature should be considered as enviable or not is open to discussion, and most certainly depends on the context. When a disease is due to the malfunction of a specific process or the lack of a specific metabolic compound, a small scope would a priori be preferable, since it guarantees that no harmful side-effects are induced by the drug. Large scopes are more difficult to assess: on the one side, a drug with a large scope is potentially more efficient in combating a multifactorial disease, as its effects can extend far beyond the immediate targets. On the other side, because it is difficult so far to quantify such large-scale effects, it cannot be excluded that they might include negative consequences for the organism. A wishful goal for the future should therefore involve moving beyond this type of qualitative view of drug-metabolism interactions towards a more quantitative view. For example, being able to predict which reactions and compounds of the metabolic drug scope are enhanced or repressed by the drug's action could lead to a whole new level of knowledge.

It is also remarkable that correlations can be observed between metabolic drug scopes and therapeutic properties of drugs. This observation strengthens the relevance of metabolic drug scopes in studying a drug's action and effects in a systems-wide context. As they discriminate the networks of action of different drugs, scopes also offer discrimination between the consequences of these actions. Whether this finding can be turned into a predictive tool, i.e. to determine scopes and drug targets with the aim of obtaining a desired effect, remains open to investigation but certainly represents a promising perspective.

## Conclusion

Metabolic drug scopes provide a new perspective on interactions between drugs and metabolic systems going beyond more traditional network-based approaches. This analysis constitutes a first step in the integration between drug-target interaction networks on the one side, and stoichiometric interactions in metabolic systems on the other. In the future, such approaches will have to be extended toward quantitative modeling, with the aim of achieving a precise understanding of how drugs interact with living systems. This knowledge will in turn enable us to improve the drug development process and create more efficient therapies based on optimized combinations of multiple drugs.

## Methods

### Construction of metabolic drug scopes

The DrugBank database is a comprehensive bioinformatics and chemoinformatics resource containing detailed information about all US approved drugs and their molecular targets [22]. We downloaded the database and identified all drugs having enzymatic targets by extracting their Enzyme Commission (EC) numbers. For each of them, we compiled the list of substrates and products using both the Kegg [26] and Brenda databases [27]. In the case of unspecific reactions having multiple substrates and products, we only selected the most common compounds, as uncommon compounds are usually not supported by the scope construction application.

For each drug, the complete set of compounds determined in this way constituted the seeds of the metabolic drug scope. Oxygen and water were added to the seeds if not already present, as it is assumed that these compounds are always available in a human metabolism context. The following cofactors were also assumed to be available for metabolic reactions, but they were not added to the seeds: ATP, ADP, NAD, NADH, NADP, NADPH, CoA. It is a recurrent question in metabolic network analysis to decide whether cofactors should be included or not. We here included all of them because the fundamental basis of the metabolic scope is to encompass the largest possible range of effects of a drug. Such effects can be transmitted by cofactors as well as by other metabolites, therefore the distinction would here appear rather artificial.

We subsequently used the MetaPath Online application [19] to construct the scopes resulting from these seeds. For each drug, we computed four different scopes based on four metabolic networks: a reference network corresponding to the union of all species vs. a human network, and for each of those a reversible vs. an irreversible topology. Scopes were saved as text files for subsequent analysis. The full set of metabolic drug scopes for 276 drugs and 4 metabolic networks is provided in Additional File 3.

It frequently occurred that multiple drugs had the same set of enzymatic targets. In this case the scopes of these drugs are necessarily identical. In several parts of our analysis such duplicate scopes were counted only once, so that we used a subset of scopes that are all distinct. A list of drugs and their enzymatic targets is provided in Additional File 4, where drugs with identical targets are highlighted in the same color.

### Clusters of metabolic drug scopes

We used the Jaccard distance between sets to define a measure of dissimilarity between scopes. For two scopes A and B, the Jaccard distance is 1 – |A∩B|/|A∪B|, where |A∩B| is the number of compounds contained in the intersection of A and B, and |A∪B| is the number of compounds contained in the union of A and B. If two scopes are identical their Jaccard distance is 0; if two scopes have no compound in common their Jaccard distance is 1.

Hierarchical clustering of metabolic drug scopes was calculated with the R statistical computing environment using the Jaccard distance as a measure of dissimilarity. At first, each observation is a small cluster by itself. Clusters are merged until only one large cluster remains which contains all the objects. At each stage the two nearest clusters are combined to form one larger cluster. We adopted the *complete linkage *method, where the distance between two clusters is computed as the distance between the two farthest objects in the two clusters. This method tends to keep outliers out of clusters and guarantees that all objects inside clusters have strongly similar characteristics. A cutoff value of 0.5 was used to define clusters, which was set by observing the clustering tree and chosen to be robust against variations.

### Consensus scopes

Following a definition set by Matthäus *et al*. [21], the *consensus scope *of a cluster is the set of compounds that are contained in at least 50% of all scopes in the cluster. Consensus scopes are useful to visualize the typical properties of a group of metabolic drug scopes. Although the threshold of 50% is arbitrary, this definition is very robust against variations of the threshold. Most consensus scopes of our clusters remain identical when the threshold varies between 30% and 90%. The same property was observed with consensus scopes of clusters of individual compounds [21].

### Therapeutic properties

The DrugBank database also contains information about the therapeutic properties and applications of each drug. This information follows the Anatomic Therapeutic Chemical (ATC) classification. The ATC system is used by the World Health Organization as an international standard for drug utilization studies. It divides drugs into different groups according to the organ or system on which they act and their chemical, pharmacological and therapeutic properties. Drugs are classified into groups at five different levels. The first level of the code consists in a letter for the anatomical group, there are 14 such groups.

The statistical significance of the enrichment of particular therapeutic classes in clusters was tested by standard chi-square tests. The null hypothesis is that the distribution of any therapy in a cluster is not different from that of the whole set of drugs. The associated *p*-values are indicated in Results.

### Scope network construction

Using the Jaccard distance as a measure of dissimilarity between scopes, we constructed a similarity network composed of scopes as nodes. Two scopes were connected by an edge if the Jaccard distance between them was higher than a given threshold. Drugs whose distance to any other drug never exceeded the threshold, which would thus constitute isolated nodes, were not included in the network representation.

### k-cores

Let *G *= (*V*, *E*) be a graph where *V *is the set of nodes and *E *is the set of edges. A subgraph *H *= (*W*, *E*|*W*) induced by the set *W *is a k-core iff ∀*v *∈ *W*, deg(*v*) > *k *and *H *is a maximum subgraph with this property [28,29]. Given a graph *G*, the algorithm of Batagelj & Zaversnik determines cores hierarchy by recursively deleting all nodes and connected edges of degree less than *k*. As a result, the remaining graph is the k-core graph.

## Authors' contributions

JMS conceived of the study, JMS and JCN performed the analyses and wrote the manuscript. All authors read and approved the final manuscript.

## Supplementary Material

Additional File 1 **Correlation between scope size and total number of drug targets**. Each sheet contains a list of drugs identified by their DrugBank accession number, the number of known targets of each drug (including non-metabolic targets) and the size of its metabolic scope. The four sheets correspond to the four metabolic networks analyzed in this paper (reference reversible, reference irreversible, human reversible, human irreversible). Click here for file

Additional File 2 **Composition of drug clusters**. Each sheet contains a list of drugs identified by their DrugBank accession number, and the identifier of the cluster each drug was assigned to. The four sheets correspond to the four metabolic networks analyzed in this paper (reference reversible, reference irreversible, human reversible, human irreversible). Click here for file

Additional File 3 **Full set of metabolic drug scopes**. This archive contains the full set of metabolic drug scopes for 276 drugs from the DrugBank database and four metabolic networks. Individual files in the archive are named according to the following nomenclature: DrugBank accession number of the drug, followed by "_hsa" for human or "_ref" for reference network, followed by "_i" for irreversible or "_r" for reversible network. Click here for file

Additional File 4 **List of drugs and their enzymatic targets sorted by EC numbers**. This sheet contains a list of drugs identified by their DrugBank accession number, and the set of known enzymatic targets of each drug identified by Enzyme Commission (EC) numbers. Drugs are sorted by increasing EC numbers, drugs sharing the same set of enzymatic targets are highlighted in color. Click here for file
